# The Bacteriological Examination of Water Supplies

**Published:** 1907-12

**Authors:** 


**The Bacteriological Examination of Water Supplies.** By WM. G. SAVAGE, M.D., D.P.H. Pp. xvi., 297. London: H. K. Lewis. 1906.

The objects of this class of book are two: first, to provide the reader with an up-to-date compilation of methods and of the facts the application of those methods have elicited; and secondly, to present the views of the author and of other observers upon the deductions which may properly be drawn from the results obtained in dealing with new material. In the present instance, as may be anticipated from Dr. Savage's wide experience of bacteriological methods, the first object is very completely attained, and the practical worker will be very grateful for the collection, in so small a compass, of all really useful existing processes for the detection and enumeration of those micro-organisms, the presence of which in potable water is a more or less conclusive indication of pollution. This portion of the book would be improved by avoidance of a good deal of repetition, which renders reference difficult, and by a more systematic display of the essential characteristics of allied species.

With the discussion, however, of the precise significance of the presence or absence of certain forms, matter of much more controversial type is introduced.

The author's attitude on this question is not a doubtful one; in parliamentary parlance he is a " whole hogger," as witness the claim that " it—*i.e.* bacteriological examination—is taking its rightful place as the most valuable of all available methods by which to judge the purity of a water supply," and the declaration that " the B. coli estimation is the essential enumeration upon which to judge the purity of waters." Do the facts justify quite so emphatic a statement ?

Whilst characterising as baseless the opinion that B. coli is ubiquitous, the writer adduces proof that it is present in the excreta of practically all vertebrates, and concludes that " obviously, with such an extensive natural habitat, this organism must needs be extensively distributed." Evidence of this is afforded by the demonstration of B. coli " in as little as 2 c.c. of upland surface waters, even from sources of undoubted purity, and away from all human or sewage pollution," and again by Dr. Houston in the water of remote Highland lochs, in which instances its presence is explained by the intervention in the one case of sheep, and in the other of trout and other " lower animals ; " an explanation that would seem to suggest that the detection of B. coli must be accompanied by an inspection of the locality before any definite conclusion can be arrived at.

It appears to be part of the case of the advocates of one kind of water examination to depreciate all other methods, and Dr. Savage does not fail to make a determined attack on chemical processes, adducing the Bridgend epidemic as a case in point. But the bearing which the source of the water has upon the selection of the appropriate method of analysis is hardly sufficiently recognised. It is, no doubt, perfectly true that a much smaller admixture of sewage with water can be recognised by bacterial than by chemical analysis, but it does not follow that the former must invariably be the best guide to the recognition of " impurity and hazard " under the conditions of natural water supplies.

When the sewage is directly introduced into and uniformly mixed with the water, and conveyed to the consumer along a capacious channel, whether superficial or subterranean, bacterial examination is, beyond doubt, the most useful aid to topographical inspection; but when the polluted water undergoes more or less perfect purification by subsoil filtration, the arrest of the micro-organisms is very capricious and uncertain, depending on the subsoil conditions of the moment, and these do not affect the presence of the perfectly characteristic soluble matters on which the chemical analysis depends. In such cases bacteriological enumeration may, and often does, afford no glimpse of the warning of danger which chemical analysis discloses with certainty.

On the whole, it may be said that Dr. Savage offers no good reason for abandoning the very generally accepted and safe position that all available means should be used simultaneously in forming an opinion on so vital a matter as the safety of a water supply, but furnishes one of the best and most useful guides to the intelligent handling of the undoubtedly important instrument so ably advocated in the work under review.

